# Laparoscopic surgery for giant retroperitoneal lymphangioma: a case report

**DOI:** 10.1093/jscr/rjaf008

**Published:** 2025-01-23

**Authors:** Shengchang Zhu, Jinggang Pan

**Affiliations:** Department of Hepatobiliary Surgery, YiChun City People's Hospital, 1061 Jinxiu Avenue, Yichun 336000, Jiangxi, China; Department of Hepatobiliary Surgery, YiChun City People's Hospital, 1061 Jinxiu Avenue, Yichun 336000, Jiangxi, China

**Keywords:** retroperitoneal lymphangioma, laparoscopic surgery, surgical treatment, minimally invasive surgery, imaging manifestation, case report

## Abstract

This case report describes a 66-year-old male diagnosed with a giant retroperitoneal lymphangioma, presenting with an abdominal mass confirmed via magnetic resonance imaging (MRI). Laparoscopic surgery was successfully performed to excise the mass, with histopathological examination confirming the diagnosis. The patient’s postoperative recovery was uneventful, with no signs of recurrence or metastasis observed at the three-month follow-up. This case underscores the feasibility and advantages of utilizing laparoscopic techniques in the management of large retroperitoneal lymphangiomas.

## Introduction

Giant retroperitoneal lymphangioma (RGL) is a rare, benign tumour that results from abnormal dilation and proliferation of lymphatic vessels [1–2]. Due to the complex anatomy of the retroperitoneum, RGLs are often significantly enlarged at the time of diagnosis, leading to symptoms such as abdominal pain, distension, and urinary obstruction. Surgical excision is generally the preferred treatment for RGLs. With advancements in minimally invasive techniques, laparoscopic surgery has become an increasingly viable method for treating retroperitoneal tumours due to its benefits of minimal trauma, reduced pain, and rapid recovery. This report details the diagnosis and treatment of a giant RGL, highlighting the value of laparoscopic surgery in managing such rare tumours and aiming to provide a reference for similar cases in the future.

## Case report

**Figure 1:**
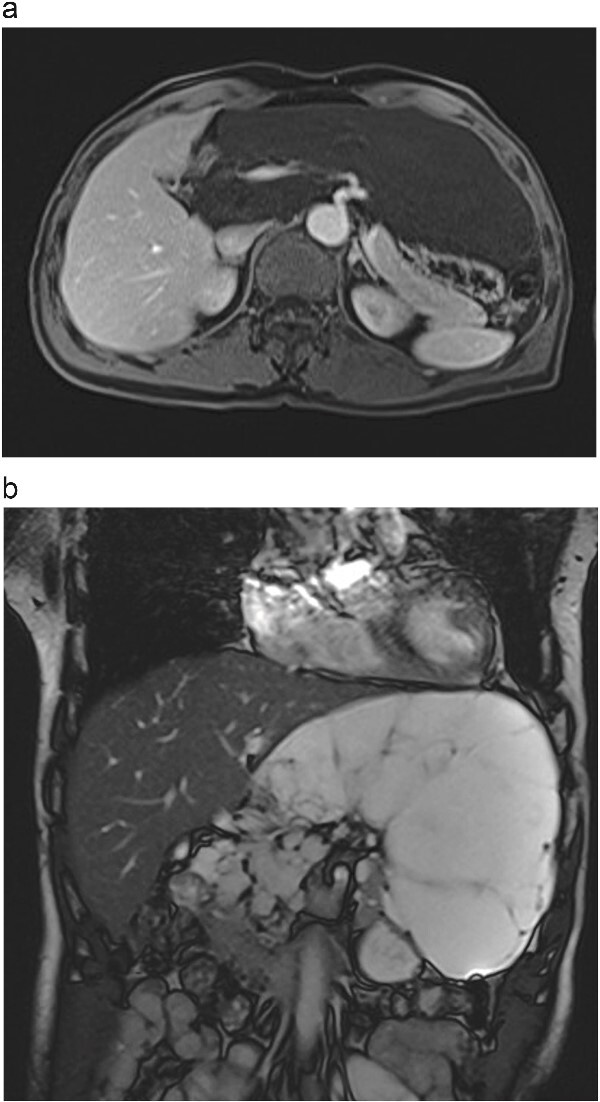
(a) Abdominal contrast-enhanced scan in the arterial phase shows the tumour compressing the portal vein, common hepatic artery, and celiac trunk. (b) T2-weighted MRI image shows the tumour with high signal intensity and no solid components.

A 66-year-old male was admitted on 15th July 2024 due to an abdominal mass detected two months earlier. Physical examination revealed a soft, non-tender abdominal mass with limited mobility in the left upper abdomen. Abdominal MRI showed a large, irregular mass in the retroperitoneal space measuring ⁓19 cm × 8 cm with clear borders and multiple internal septa. The mass was compressing surrounding structures, including the portal vein, common hepatic artery, and coeliac trunk (Fig. 1a and b).

**Figure 2:**
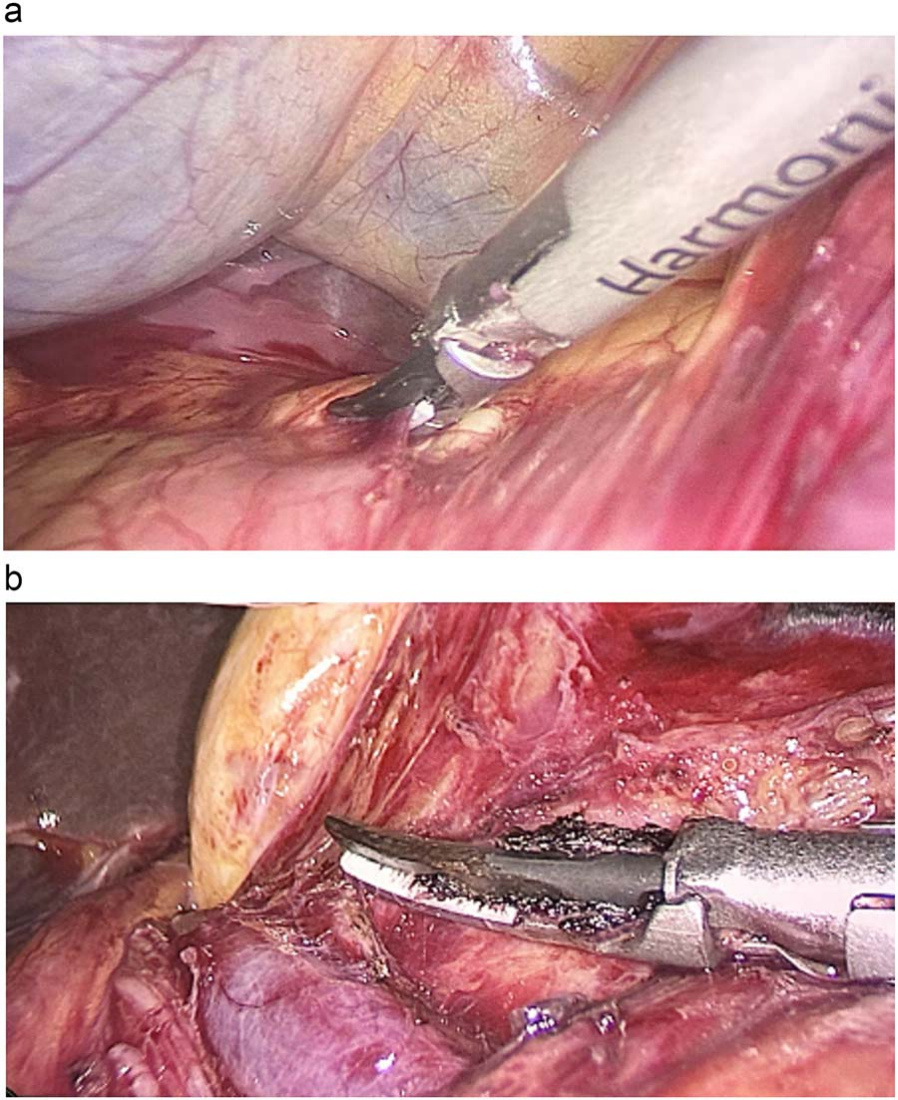
(a) Intraoperative view shows the tumour extending beyond the right side of the duodenal ligament. (b) Laparoscopic dissection of the tumour from the portal vein.

**Figure 3:**
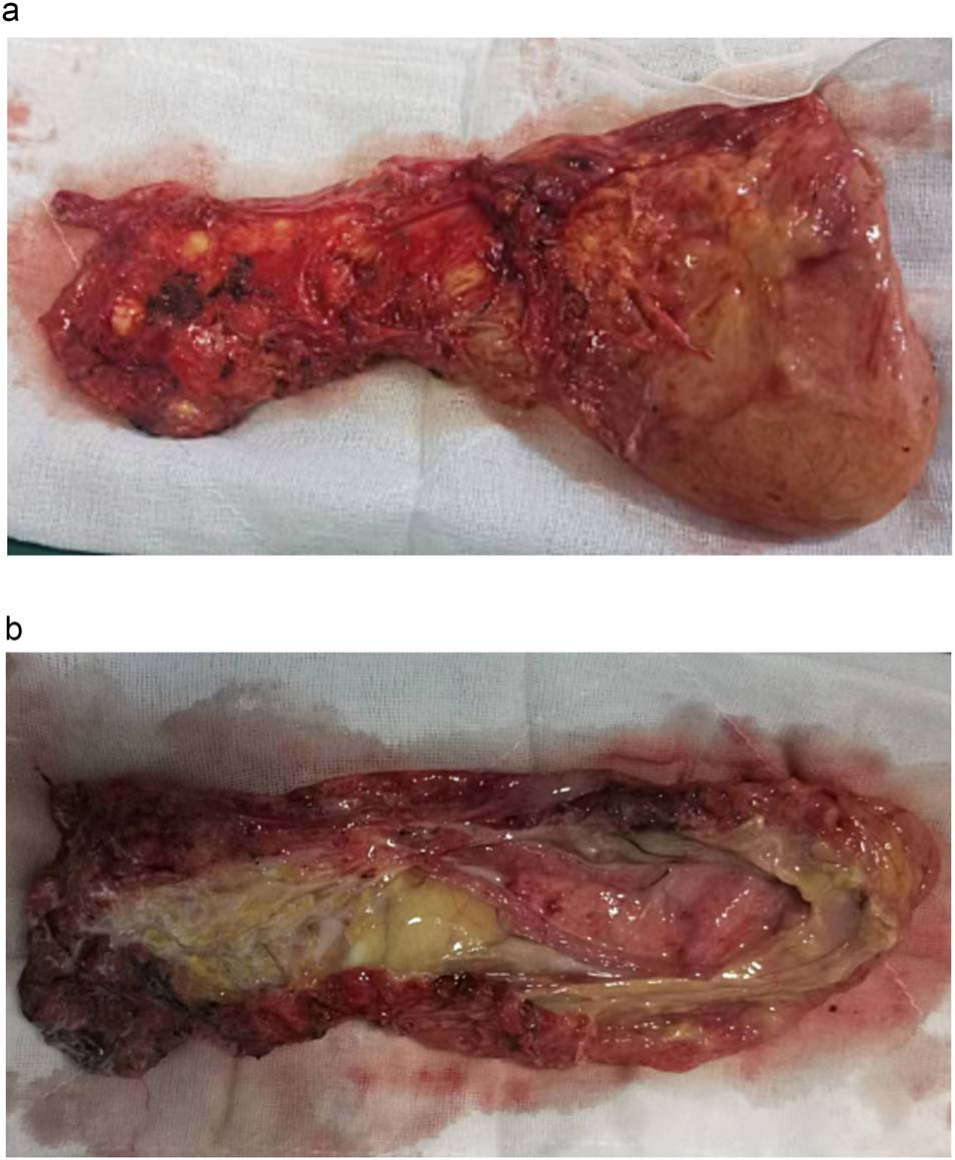
(a) The tumour is completely excised. (b) The tumour is cut open, revealing milky white fluid and a small amount of fatty tissue.

On 19th July 2024, the patient underwent laparoscopic resection of the giant retroperitoneal lymphangioma. Intraoperatively, a large cystic mass was found protruding from the omental bursa, with additional cysts observed on the right side of the hepatoduodenal ligament, indicating a retroperitoneal origin (Fig. 2a and b). Due to the large size of the cyst, a portion of the cystic fluid was aspirated to create space for the procedure. The cyst wall was carefully dissected and separated from surrounding structures, including the common hepatic artery, left gastric artery, the superior border of the pancreas, and the portal vein, enabling complete excision (Fig. 3a).

**Figure 4:**
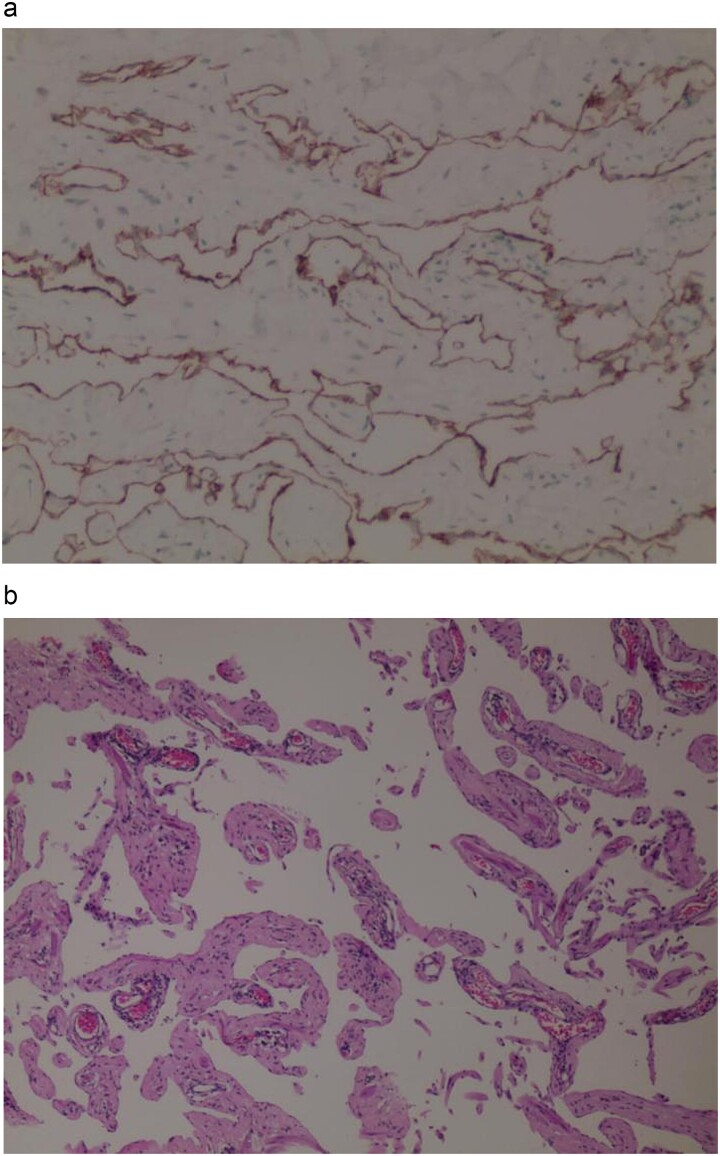
(a) Immunohistochemistry D2–40 (+). (b) Haematoxylin and eosin staining.

Postoperative histopathological examination confirmed the diagnosis of lymphangioma. Macroscopically, the tumour appeared as a fatty cystic mass measuring 18.5 × 8 × 4 cm, with a multi-locular, honeycomb-like appearance containing milky white fluid (Fig. 3b). Immunohistochemistry results were positive for D2–40 and CD31, confirming the lymphatic nature of the tumour (Fig. 4a and b). The patient was discharged one week postoperatively, and a follow-up CT scan three months later showed no signs of tumour recurrence or metastasis.

## Discussion

Giant retroperitoneal lymphangiomas are rare, benign tumours with an unclear pathogenesis. Lymphangiomas are primarily caused by abnormal proliferation of lymphatic vessels and can be classified histopathologically into simple lymphangiomas, cavernous lymphangiomas, and cystic lymphangiomas, with the latter being the most common [3]. Cystic lymphangiomas are characterized by more pronounced dilation of lymphatic vessels and are usually diagnosed during childhood, although cases in adults have also been reported [4–6].

Retroperitoneal lymphangiomas are benign tumours, and surgical excision of the lesion generally ensures a favourable prognosis. Postoperative treatment is typically unnecessary, but regular follow-up and monitoring are recommended to detect potential recurrence or complications.

Although lymphangiomas exhibit diverse clinical manifestations, giant retroperitoneal lymphangiomas often cause abdominal discomfort, pain, and other compressive symptoms due to their large size, adding to the complexity of diagnosis and treatment.

Laparoscopic surgery, a minimally invasive technique, aligns with the principles of Enhanced Recovery After Surgery (ERAS) by offering advantages such as reduced trauma, quicker recovery, and less postoperative pain. This approach is becoming increasingly popular in the treatment of giant retroperitoneal lymphangiomas. However, the complex anatomy of the retroperitoneum and the proximity of these tumours to major blood vessels and vital organs present significant challenges, including a higher conversion rate to open surgery and an increased risk of intraoperative and postoperative complications [7–8]. Therefore, it is essential that laparoscopic treatment of giant retroperitoneal lymphangiomas be performed in centres with advanced laparoscopic expertise.

In this case, preoperative CT revealed a tumour measuring ⁓19 cm in its largest diameter, located deep within the retroperitoneum and closely adjacent to the portal vein, common hepatic artery, and coeliac trunk. The tumour’s complex location posed significant surgical challenges. However, with detailed preoperative preparation and meticulous surgical planning, the tumour was successfully excised laparoscopically. The use of laparoscopic techniques in this case demonstrated advantages such as minimal blood loss and rapid postoperative recovery, highlighting the potential benefits of laparoscopic surgery in treating giant retroperitoneal lymphangiomas.

Despite these advantages, laparoscopic surgery for RGLs also presents certain challenges [9]. The complex anatomy of the retroperitoneum, with numerous vital blood vessels and organs in close proximity, increases the difficulty and risk of surgery. Complete excision of a large lymphangioma requires precise surgical techniques to avoid intraoperative rupture and reduce the risk of postoperative recurrence. In this case, careful dissection and appropriate intraoperative techniques were employed to prevent such complications.

Postoperative management is crucial to the success of surgery. Close monitoring of the patient’s vital signs and abdominal symptoms is necessary to promptly identify and address any potential complications. In this case, the patient’s postoperative recovery was uneventful, demonstrating the effectiveness and importance of proper postoperative care.

## Conclusion

Laparoscopic surgery, as a minimally invasive technique, has shown significant advantages in treating giant retroperitoneal lymphangiomas. The success of this case suggests that with detailed preoperative evaluation and precise intraoperative techniques, laparoscopic surgery can safely and effectively achieve complete resection of giant retroperitoneal lymphangiomas. Further large-scale studies are needed to evaluate the long-term efficacy and safety of this approach, providing the best treatment options for more patients.

## Conflict of interest statement

None declared.

## Funding

None declared.
